# Management of *Rumex dentatus* L. in *Vicia faba* L. cultivations via *Ononis vaginalis* Vahl. As a potential bioherbicide

**DOI:** 10.1016/j.heliyon.2024.e26381

**Published:** 2024-02-18

**Authors:** Roufaida M. Elghobashy, Shimaa A. Rashed, Amal M. Fakhry, Reham M. Mostafa, Heba S. Essawy, Salama M. El-Darier

**Affiliations:** a Biology and Geology Department, Faculty of Education, Alexandria University, Alexandria, Egypt; b Botany and Microbiology Department, Faculty of Science, Alexandria University, Alexandria, Egypt; c Botany Department, Faculty of Science, Benha University, Egypt

**Keywords:** Allelopathy, Bioherbicides, *Ononis vaginalis*, Phytochemistry, Weed management

## Abstract

Biological control of undesirable weeds associated with crop cultivation is a sustainable approach that can reduce chemical herbicide dependence. The current study aimed to assess the bio-herbicidal potential of the donor species *Ononis vaginalis* Vahl. on germination efficiency as well as various growth and physiological parameters of the recipient species *Rumex dentatus* L., a major broad bean pest (*Vicia faba* L.). To assess the greatest inhibitory allelopathic effect on the recipient species in mixed (*Rumex dentatus* L*.* and *Vicia faba* L.) and pure cultures (each one separately), two experiments were conducted under laboratory conditions. A Petri dish experiment using *O. vaginalis* shoot aqueous extract (5%, 10%, 20%, and 40%) and a pot experiment using *O. vaginalis* shoot crude powder (1%, 2%, 5%, and 10%) were conducted to investigate its biological activity on some growth and physiological parameters of both crop and weed species. *O. vaginalis* underwent a general phytochemical screening that revealed a high production of allelochemicals, which are secondary metabolites and may have a function like that of natural herbicides. The result showed that the germination of *V. faba* seeds in both pure and mixed cultures was not significantly affected by low levels of *O. vaginalis* shoot aqueous extract treatments in pure and mixed cultures, in contrast, those recorded for *R. dentatus* gradually dropped as levels of O. vaginalis increased in both cultures. Results recorded a significant increase in total phenolics of *V. faba* shoots and roots under different treatments, except at the high concentrations of crude powder at the donor species level (5 and10%). A reduction in the total phenolic and flavonoid fractions was observed in *R. dentatus* roots under varying concentration treatments. Conversely, under high concentration treatments, flavonoids decreased in the roots of the mixed culture of *R. dentatus* but increased in the shoots. In conclusion, allelopathy can be used to suppress weeds in field crops. The study confirmed the use of *O. vaginalis* into current weed control techniques. *O. vaginalis* could be explored further for weed suppression in the field.

## 1 Introduction

Weeds have been considered one of the most detrimental aspects to agricultural output, producing a significant loss in yield through direct competition for resources [1]. Damages caused by weeds are far greater than damages from any category of agronomic pests [2]. In this environment, effective weed management is crucial to minimize weeds' negative impact on crop yield [3]. The use of synthetic herbicides has enhanced crop production and productivity by reducing weed infestation, but it has also introduced several environmental risks. To avoid the negative impacts of synthetic herbicides and alleviate social health problems, bioherbicides are a preferable option to replace chemical herbicides [4,5].

Allelopathy is a possible method for weed management, presenting an environmentally beneficial alternative to synthetic herbicides [6]. It involves the use of chemical compounds released by plants to limit the germination and growth of weeds [7]. By identifying and quantifying these natural weed control compounds (allelochemicals), it is possible to develop new natural herbicides [8]. Bioherbicides are one of the most effective weed control solutions for sustainable agriculture. they are typically generated from plants contain phytotoxic allelochemicals that can control weed populations. Bioherbicides have shown considerable potential in suppressing weed seed germination and growth [9,10].

Allelopathic Plants that release allelochemicals into the environment are biodegradable [11], hence producing less pollution. It can be used to control weeds by using novel natural products (mainly allelochemicals) as natural bio-degradable herbicides or by directly utilizing natural allelopathic interactions [12]. Under sustainable agriculture, the application of allelopathic and medicinal plants has been proposed as a workable alternative for weed control [13]. Previous research has demonstrated the value of allelopathic plants for long-term agricultural productivity [14] and Their products have drawn a lot of attention because they show off their herbicidal, antifungal, insecticidal, and pesticidal qualities [15,16]. Overall, bioherbicides shows promise as a sustainable and environmentally friendly method for weed management in agriculture.

*Ononis vaginalis* Vahl. (Fabaceae) is a perennial subshrub belonging to Fabaceae and is common on maritime sand [17]. The plant is a near-endemic species in Egypt and is very common along the eastern side of the Mediterranean coastal belt [18,19]. This species is widely used to treat several diseases [20]. The roots of *Ononis vaginalis* have been found to contain compounds with antiviral activity and cytotoxicity. It contains bioactive compounds. Bioactive alkyl resorcinols, spinonin, have recently been isolated from Ononis species [21,22]. *O. vaginalis* flowers have been used to isolate Flavonoids and norphenylpropanoid glucosides [23,24]. Additionally, saponins from Passiflora alata and Quillaja saponaria, which are related species, have shown anti-Trichomonas vaginalis activity [25]. However, there is no specific mention of Ononis vaginalis being used as a bioherbicide in the abstracts provided. Further research may be needed to determine the effectiveness of Ononis vaginalis as a bioherbicide.

*Vicia faba* L. (Fabaceae) is considered an economically important legume crop worldwide [26]. It is among the healthiest staple foods in the Egyptian diet and provides the majority of necessary macronutrients [27]. *Rumex dentatus* L. (polygonaceae) is an annual that grows extensively throughout Egypt, Europe and Asia. It occurs in waste places, shores, canal banks, and cultivated fields [28]. *R. dentatus* is one of the dominant weeds in many crops [29]. Due to its high infestation rate and fierce competition, this weed can seriously reduce yields [7]. It is a problematic weed that requires effective management strategies. Manual and mechanical methods are becoming costly, while cultural practices are not always effective due to the weed's adaptability. Chemical herbicides have been used, but they have limitations and associated problems [30].

The aim of the current study was to assess *O. vaginalis*, a donor species, and *R. dentatus*, a recipient species, for bioherbicidal potential relative to germination efficiency, some growth parameters, as well as some metabolic changes in both crop and weed species. Various concentrations of *O. vaginalis* aqueous extract and crude powder were tested for the suppression of *R. dentatus* growth in both pure and mixed cultures.

## 2 Materials and methods

### 2.1 Collection of plant materials

Donor species (*O. vaginalis*) were taken during the vegetative and flowering stages in summer 2021 from sand dunes habitat in the northwest of Egypt in Matruh governorate. The plant materials were air-dried and cut into small pieces then ground to powder and sieved. Seeds of the crop species (*V. faba*) were purchased from the International Research Center, El-Dokki, Giza, Egypt. Healthy, uniform seeds of *R. dentatus* were collected from broad bean fields in El-Bihara province during June 2021. The formal identification of the plant materials was carried out in the Herbarium of the Faculty of Science, Alexandria University.

### 2.2 Preparation of donor species aqueous extracts

Stock aqueous extracts and subsequent dilutions were prepared by extracting 100 g of dry powder from the donor species with 1000 ml of distilled water. This was the full-strength concentration (100%). The extracts were prepared no more than 48 h before use and stored in a refrigerator at 5 °C until required. The purified extract was adjusted to pH 6.8 with 1 M HCl. At such an optimal pH, no significant growth inhibition occurred [12,31]. A series of dilutions were made from the stock solutions (5%, 10%, 20%, and 40%, in addition to the control) [32].

### 2.3 General phytochemical screening of the donor species

#### 2.3.1 Steroids and terpenoids

Qualitative phytochemical screening to determine steroids and terpenoids was carried out according to Ref. [33]; five ml of the extract was mixed with 2 ml of chloroform and concentrated sulphuric acid to construct a layer. A reddish-brown color of the interface indicated the presence of steroids and terpenoids.

#### 2.3.2 Carbohydrates and glycosides

Approximately 1 g of dried plant powder extracted with 10 ml ethanol (50%) and filtered. The ethanolic extract was combined with 0.5 ml alpha naphthol reagent, and then 1 ml sulphuric acid was gently poured onto the test tube's wall. A violet ring appeared at the interface, indicating the presence of carbohydrates or glycosides [34].

#### 2.3.3 Tannins

Tannins were also detected, according to Ref. [33], as about 0.5 mg of crude plant powder was boiled in 20 ml of water in test tubes and filtered. The addition of a few drops of 0.1% ferric chloride produced brownish-green or blue-black coloration, indicating the presence of tannins; however.

#### 2.3.4 Alkaloids

Alkaloids were estimated as follow; 1-g of crude plant powder was taken in a conical flask, and 3 ml of ammonia solution was added. It could stand for a few minutes to assess the free alkaloids. 10 ml of chloroform was added to the conical flask shaken by hand and then filtered. The chloroform was evaporated from the crude extract in a water bath and 3 ml of Mayer's reagent was added. Quick cream-coloured precipitation was found, indicating the presence of alkaloids [33].

#### 2.3.5 Flavonoids

Flavonoids were assessed according to Ref. [33]; Approximately 1 g of dried plant powder was extracted with 10 mL of 50% ethanol and filtered. Five ml of the extract were made alkaline by adding 10% sodium hydroxide solution. The appearance of yellow suggests the presence of flavonoids. Five ml of the extract was combined with 1 ml of concentrated hydrochloric acid, and magnesium turnings were added. Red colour suggested the presence of flavonoids [33].

#### 2.3.6 Saponins

Saponins were distinguished according to Ref. [35], by putting 1 ml of the stock solution of the plant extract in a test tube and dilution with 20 ml of distilled water. It was shaking by hand for 15 min. A foam layer formed on top of the test tube. This foam layer suggested the presence of saponins, High-performance liquid chromatography was performed utilizing the analytical HPLC system [36].

#### 2.3.7 In vitro bioassay

A Petri dish experiment was conducted to study the biological impact of *O. vaginalis* shoot aqueous extract (OVSAE) on the percentage of germination (GP%), plumule (PL) and radicle (RL) lengths of both crop (*V. faba***)** and weed (*R. dentatus*) species. 10 seeds of each of the weed and crop species were placed in 9-cm diameter Petri-dishes coverd with two discs of Whatman No.1 filter paper under normal laboratory conditions with day temperatures ranging from 22 to 27 °C and night temperatures from 14 to 18 °C. Fifteen ml of the respective donor species aqueous extracts (5, 10, 20 and 40%) or distilled water as a control were added daily to three replicates in a randomized complete block design. Before sowing, the seeds of the two-recipient species were immersed in 2% CHLOREX for 2 min then rinsed four times with distilled water [37].

The inhibition percentage (IP) of the donor species extracts was expressed as a percentage of the growth (germination) of the test species at different concentration levels with respect to water control. using the following formula.

IP% = 100 – (E2 × 100/E1) Where, IP = % inhibition. E1 = Response of control plant. E2 = Response of treatment plant.

#### 2.3.8 Pot bioassay

A pot experiment was conducted to assess the effect of various levels (1%, 2%, 5%, and 10%) of *O. vaginalis* air-dried shoot crude powder (OVSCP) on some growth parameters such as seedling shoot and root lengths (cm), seedling fresh and dry weights (g) as well as some physiological parameters such as total proteins, carbohydrates, phenolics and flavonoids of crop species.

Ten seeds of the two recipient species were sown in plastic pots (16 cm in diameter and 15 cm height) with approximately 1000 g of clay loam soil (pH: 7.7, N:1.1 mgg^−1^, P: 0.5 mgg^−1^ and K: 3.5 mgg^−1^) and completely mixed (w/w) with 1, 2, 5 and 10% of crude powder of the donor species, in addition to control treatment (no donor species). The treatments were distributed in a totally randomized block design with three repetitions. The plants were watered every two days with filtered tap water, with the amount of water equal to the average soil-plant evapotranspiration derived from weight loss over a 24-h period. The experiment was conducted under standard laboratory settings (day temperature 22–27 °C, night temperature 14–18 °C, relative humidity 75 ± 2%, and 12/10 h light/dark photoperiod). After 21 days, the homogeneous seedlings were harvested [37].

### 2.4 Determination of some physiological parameters

#### 2.4.1 Total available carbohydrates

The extraction and estimation of total available carbohydrates (TAC) were indicated by Ref. [37], An aliquot (100 mg) of finely powdered oven-dry plant material was added to a boiling tube. 10 ml of 0.7 N HCl were then added to the tube, which was placed in a boiling water bath for 30 min. The hydrolysate was neutralized to a phenol red endpoint and diluted to a specified volume. In a test tube, a 2-mL aliquot of carbohydrate solution was combined with 1 ml of a 5% aqueous phenol solution. Following that, 5 cc of concentrated sulphuric acid was quickly added to the mixture. After standing for 10 min, the test tubes were vortexed for 30 s before being placed in a room-temperature water bath for 20 min to develop colour. Then, a spectrophotometer was used to measure light absorption at 490 nm.

#### 2.4.2 The total protein fractions

The complete protein fraction was extracted by adding 10 ml of 0.5 N NaOH to about 100 mg of oven-dried plant material and leaving it overnight. The extract was finished to volume with distilled water [38]. Estimation of total protein was done as the method described by Ref. [38]; 1 ml of the plant extract was combined with 0.9 ml of reagent A (2 g potassium sodium tartrate and 100 g sodium carbonate dissolved in 1 L of 0.1 N sodium hydroxide) and incubated for 10 min in a water bath at 50 °C. After cooling to room temperature, 0.1 ml of reagent B (2 gm potassium sodium tartarate + 1 gm CuSO4.5H2O dissolved in 100 ml of 0.1 N sodium hydroxide) was added, thoroughly mixed, and the tubes left to stand for 10 min 3 ml of reagent C (one volume of Folin-Ciocalteau reagent (BDH) diluted to ten volumes with distilled water) were quickly added and mixed with a vortex mixer. The tubes were then incubated at 50 °C for 10 min.

#### 2.4.3 Total phenolic contents

The total phenolic content of the extracts was measured using the Folin-Ciocalteu reagent technique, as reported in Ref. [39]. To summarize, an aliquot of the extracts was combined with 5 ml of Folin-Ciocalteu reagent (previously diluted with water 1:10 v/v) and 4 mL (75 g/l) of sodium carbonate. The tubes were vortexed for a few seconds before being let to stand at 26 °C for 30 min to develop the colour. The absorbance of samples and standards was measured at 765 nm on a Shimadzu 210A double beam spectrophotometer. The total phenolic content was reported as gallic acid (mg/g dry weight).

#### 2.4.4 Total flavonoid contents

The total flavonoid content of standards and samples was determined using the aluminium chloride colorimetric assay technique [40] Briefly, 0.5 ml of 2% AlCl_3_ ethanol solution was mixed with 0.5 ml of sample solution. After 1 h at room temperature, the absorbance was measured at 420 nm. A golden colour showed the presence of flavonoids. Total flavonoid content was estimated using quercetin (mg/g d.w.).

### 2.5 Statistical analysis

The data was analyzed using a one-way ANOVA [41]. Means were compared pairwise using the least significant difference (LSD) at a probability of 0.05. The figures were displayed using Tukey's test with 5% probability.

## 3 Results

### 3.1 General phytochemical screening of the donor species

General phytochemical screening showed an abundant production of secondary metabolites with remarkable variation, as detailed in Tables (1) and (2). The maximum presences were recorded for tannins and high concentrations of saponins, steroids, flavonoids, and essential oils. Moderate concentrations were for alkaloids, terpenoids, and glycosides (Table 1.).

**Table 1:** Qualitative phytochemical screening of *O. vaginalis.*

Constituent	AlK	SAP	TRP	TAN	FLV	STE	ESO	GLC
Rank	++	+++	++	++++	+++	+++	+++	++

ALK: Alkaloids, SAP: Saponins, TRP: Terpenoids, TAN: Tannins, FLV: Flavonoids, STE: Steroids, ESO: Essential oils, GLC: Glycosides. Very important presence ++++, Important presence +++, Average presence ++, Weak presence +.

Analytical HPLC system for phenolic metabolites showed that qualitative and quantitative differences were detected in phenolic profiles (Table 2). HPLC data reported the presence of eight phenolic compounds, the major compounds are gallic acid, chlorogenic acid, caffeic acid, 3,5- dicaffeoyl quinic acid, 4,5-dicaffeoyl quinic acid, rutin, quercetin and cinnamic acid. The most abundant peak in the methanol extracts was identified as rutin (38.7%) followed by 3,5- dicaffeoyl quinic acid (22.8%) and caffeic acid (21.29%).

**Table 2:** **HPLC data of the phenolic compounds detected in the methanol extracts of *O. vaginalis* and detected by UV absorbance at 320 nm.** in mg/g of dry plant material.

Peak Name	RT (min)	Area (mAU)	Amt/Area (%)	Amount (mg/g dwt)
Gallic acid	2.340	3034.634	0.110	4.534
Chlorogenic acid	2.355	5082.431	6.250	31.384
Caffeic acid	2.881	1.342	1.204	141.497
3,5- Dicaffeoyl quinic acid	3.332	7655.943	1.714	152.054
4,5-Dicaffeoyl quinic acid	3.530	9416.352	7.013	66.033
Rutin	4.524	4448.152	1.283	257.735
Quercetin	9.767	124.117	6.247	7.8222
Cinnamic acid	13.334	2628.426	1.237	3.452

**RT**: Retention time, **mAU**: milli-absorbance unit.

### 3.2 Germination bioassay

**Fig. 1:**
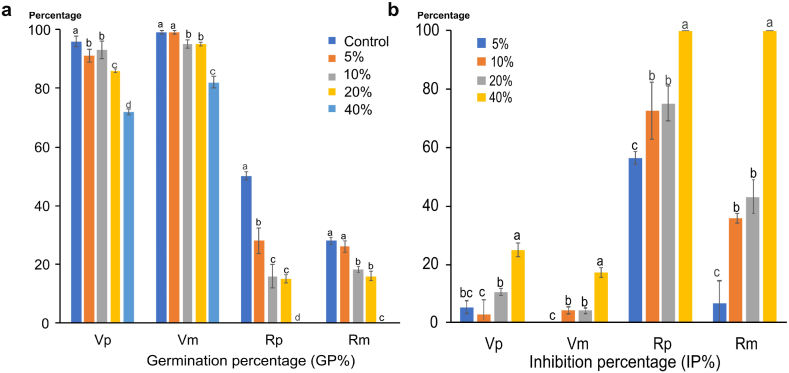
Allelopathic effect of *O. vaginalis* shoot aqueous extract on a: germination percentage (GP%) and b: inhibition percentage (IP%) of *V. faba* and *R. dentatus* seeds in pure and mixed cultures. Vp: *V. faba* (pure) **Vm:***V. faba* (mixed) **Rp:***R. dentatus* (pure) Rm**:***R. dentatus* (mixed) The results are the mean (n = 3). Columns with same letters are not significantly different at p value 0.05 using a Tukey's test at 5% of probability, figures expressed as mean ± standard error.

Data in Figure (1 (a,b)) indicate that the growth of *R. dentatus* is significantly reduced under all treatments in both pure and mixed cultures (p < 0.05). The degree of inhibition on the growth of *R. dentatus* is largely dependent on the concentration of OVSAE. The germination percentage in *R. dentatus* was completely inhibited at 40% concentration treatment in both pure and mixed cultures. On the other hand, *V. faba* was less affected by the different treatments.

**Fig. 2:**
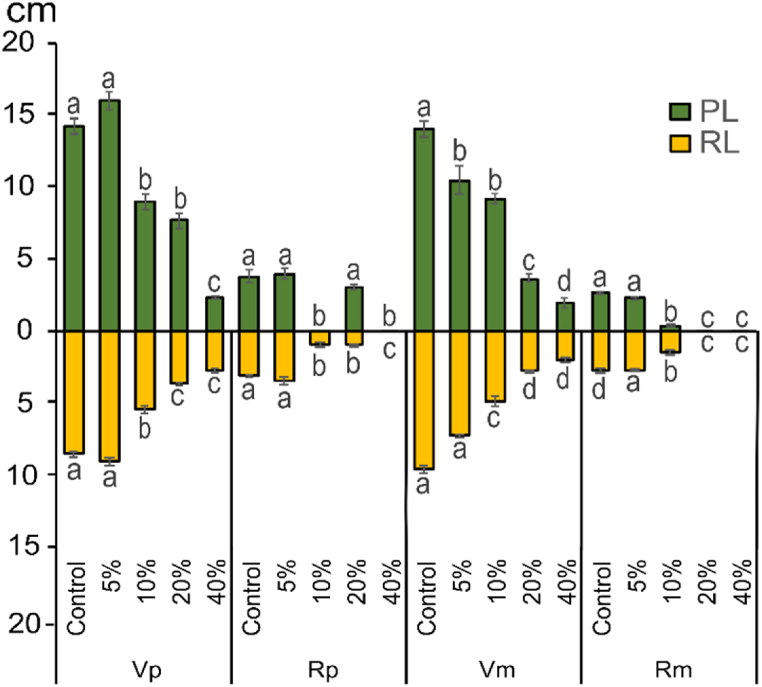
Allelopathic effect of *O. vaginalis* shoot aqueous extract in plumule (PL) and radicle (RL) lengths (cm) of *V. faba* and *R. dentatus* in pure and mixed cultures. **Vp:***V. faba* (pure) **Vm:***V. faba* (mixed) Rp**:***R. dentatus* (pure) Rm**:***R. dentatus* (mixed) Columns with same letters did not differ between treatments using a Tukey's test at 5% of probability, figures expressed as mean ± standard error.

The effects of different concentrations of OVSAE on the plumule and radicle length of *V. faba* and *R. dentatus* seedlings in pure and mixed cultures are shown in Figure (2). The plumule and radicle lengths of both the crop and weed species were significantly (p ≤ 0.05) reduced gradually in response to different concentrations of OVSAE, in mixed culture treatments. However, a slight increase is recorded in the radicle and plumule lengths of both *V. faba* and *R. dentatus* in pure culture under the lowest concentration (5%) of OVSAE treatment.

### 3.3 Growth bioassays

**Fig. 3:**
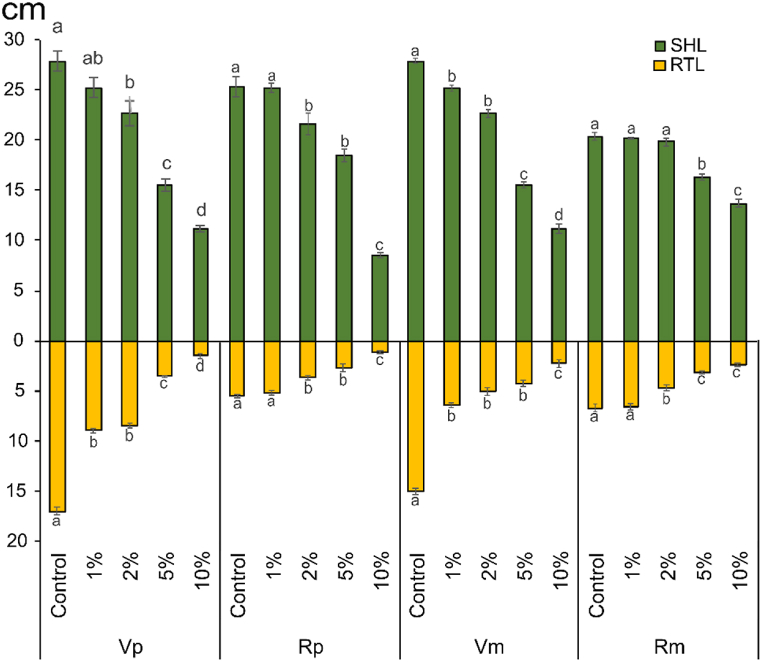
Allelopathic effect of *O. vaginalis* shoot crude powder on shoot length (SHL) and root length (RTL) (cm) of *V. faba* and *R. dentatus* in pure and mixed cultures. **Vp:***V. faba* (pure) **Vm:***V. faba* (mixed) **Rp:***R. dentatus* (pure) Rm**:***R. dentatus* (mixed) Columns with same letters did not differ between treatments using a Tukey's test at 5% of probability, figures expressed as mean ± standard error.

Generally, the lengths of shoots and roots of both *R. dentatus* and *V. faba* showed a gradual decrease with increasing concentrations of OVSCP in both pure and mixed cultures (Fig. 3). The highest reduction in *R. dentatus* shoot (56.1, 32.5%) and root (79.7 and 64.2%) in both pure and mixed cultures, respectively, was recorded at 10% relative to the control. It is notable that the root system is more sensitive to OVSCP, showing a higher length reduction than shoot lengths.

**Fig. 4:**
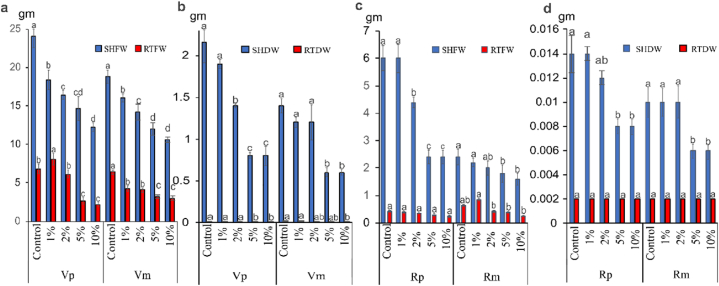
Allelopathic effect of *O. vaginalis* shoot and root crude powder (a) fresh (SHFW, RTFW) and (b) dry (SHDW, RTDW) weight (g) of *V. faba* respectively and (c& d) *R. dentatus* in pure and mixed cultures. Data are means of three replicates. **Vp:***V. faba* (pure) **Vm:***V. faba* (mixed) **Rp:***R. dentatus* (pure) **Rm:***R. dentatus* (mixed). Columns with same letters did not differ between treatments using a Tukey's test at 5% of probability, figures expressed as mean ± standard error.

The accumulation of dry matter in *V. faba* was reduced with increasing concentrations of OVSCP. Generally, the root system seems to be less affected and shows slight reductions with increasing concentrations of OVSCP (Fig. 4(a–d)).

### 3.4 Determination of some physiological parameters

**Fig. 5:**
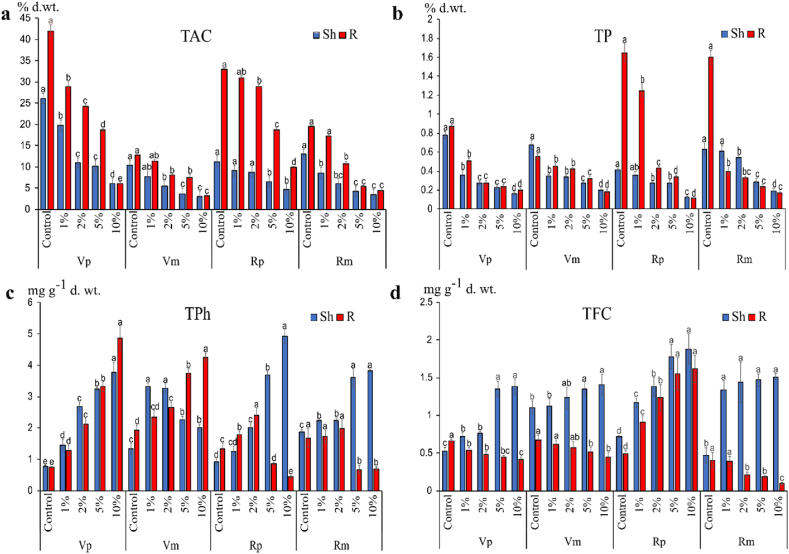
Allelopathic effect of *O. vaginalis* shoot crude powder on (a) total available carbohydrates (TAC) (% d.wt.), (b) total proteins (TP) (% d.wt.), (c) total phenolics (TPh) content (mg g^−1^ d. wt.) and (d) total flavonoids content (TFC) (mg g^−1^ d. wt.) in shoot and root of *V. faba* and *R. dentatus* in pure and mixed cultures. Data are means of three replicates. Vp: *V. faba* (pure) Vm: *V. faba* (mixed) Rp: *R. dentatus* (pure) Rm: *R. dentatus* (mixed). Columns with same letters did not differ between treatments using a Tukey's test at 5% of probability, figures expressed as mean ± standard error.

The variation of different chemical constituents in both *V. faba* and *R. dentatus* in pure and mixed cultures is illustrated in Fig. 5(a–d). Generally, data showed that total available carbohydrates (TAC) and total proteins (TP) significantly (p < 0.05) decreased in response to different concentrations of OVSCP in both pure and mixed cultures.

On the other hand, data showed that total phenolics (TPh) content increased in *V. faba* shoot and root, except in high concentration treatments, where the amount of phenolic compounds decreased in the shoot of the mixed culture. In *R. dentatus*, TPh content increased significantly in shoots in both pure and mixed cultures, while it decreased in roots under high concentration (5 and 10% OVSCP) treatments (Fig. 5 c). Generally, the amount of total flavonoid content (TFC) increased in shoots but decreased in roots of *V. faba* with increasing concentrations of OVSCP in both pure and mixed cultures. On the other hand, the TFC showed an increase in shoots of *R. dentatus,* with increasing concentrations of OVSCP in both cultures. This coincided with a notable significant reduction of TFC in roots of the weed species in pure culture, and a considerable increase in mixed culture (Fig. 5 d).

## 4 Discussion

Chemical herbicides pose serious health and environmental risks [42]. As a result, it is vital to propose technologies that not only lower production costs but are also environmentally benign [43]. Allelochemical use in agricultural systems can help to achieve a long-term and integrated pest management approach [12,44,45]. It has been demonstrated that several plant species are now crucial as unique weed control agents [46]. Allelochemicals are capable of suppressing the germination and growth of several weeds. In the present study, it was proved that shoots of *O. vaginalis* have a potential bio-herbicidal activity on *R. dentatus* in broad bean (*V. faba*) cultivations, as the seed germination was significantly decreased by increasing the *O. vaginalis* shoot aqueous extract, while *V. faba* was much less affected. Similar results confirmed that high concentrations of *O. vaginalis* retard the growth of lettuce [47]; related to the repression of cell division by gibberellin and indole acetic acid. The root length was significantly shorter than the shoot length. It was realized that the substantial inhibitory action of *O. vaginalis* on root elongation, as compared to shoots, would be owing to direct contact of the root with the extract or crude powder, and then with inhibitory compounds [48].

The results showed that the application of OVSCP resulted in a considerable reduction in total available carbohydrates and total proteins. The reduction protein content could be attributed to the accumulation of phenolic glycine, which can interfere with cytoplasmic ribosomes and RNA formation, reducing protein synthesis [[49], [50], [51]]. Phenolic compounds are some of the most important and widespread plant allelochemicals in the ecosystem. Total phenolic content showed a significant increase in *V. faba* shoots and roots except in high concentration treatments, where the number of phenolic compounds decreased in the shoots of mixed culture. Total phenolics content in *R. dentatus* increased significantly in shoots in both pure and mixed cultures, while it decreased in roots under higher concentrations (5 and 10% OVSCP) treatments. Similarly, the allelopathic effect of *Alhagi maurorum* reflected a significant reduction in total phenolic content in pea shoots as compared with the control plant [52].

In both pure and mixed culture treatments, the total flavonoid content increased in the shoots but decreased in the roots of *V. faba* with increasing concentrations of OVSCP. In *R. dentatus*, total flavonoid content increased in the shoots of both pure and mixed cultures, while it decreased in the roots of mixed cultures at high concentrations. The flavonoid is often used to describe a wide range of natural compounds with a C6–C3–C6 structure [53]. Flavonoids have antioxidant properties through a variety of processes, including scavenging free radicals and regulating the enzymes that produce free radicals [54].

In conclusion, the paper assesses O. vaginalis' bio-herbicidal potential against *R. dentatus*, a serious pest of *V. faba* cultivations. The study verifies the incorporation of *O. vaginalis* into existing weed control management systems, implying its potential as a natural herbicide for suppressing *R. dentatus*. General phytochemical screening of *O. vaginalis* reveals significant production of allelochemicals as secondary metabolites, which may act as natural herbicides. Low concentrations of *O. vaginalis* had no noticeable effect on *V. faba* but did result in a steady decline in *R. dentatus* seed germination. The study also looks at how total phenolics and flavonoids alter in the shoot and root of *V. faba* and *R. dentatus* when treated with various concentrations of *O. vaginalis*.

The effectiveness of weed control may be increased by encouraging businesses to develop herbicides based on allelochemicals and comprehending the mechanism of action of allelochemicals. The present study recommended the integration of allelopathy for the control of *R. dentatus* in weed management systems. Further experiments on *O. vaginalis* could be carried out to test its potential use for suppressing weed growth under field conditions and investigating the long-term and exploring the specific mechanisms by which *O. vaginalis* allelochemicals inhibit the growth of *R. dentatus* and their potential impact on non-target organisms would enhance understanding. Conducting studies to assess the effectiveness of *O. vaginalis* in different agricultural systems and regions would help determine its broader applicability. Investigating potential interactions between *O. vaginalis* and other weed species would provide a more comprehensive understanding of its herbicidal properties.

## Funding

No funding was received for conducting this study.

## Data availability

All data generated or analyzed during this study are included in this published article.

## Ethics approval and consent to participate

There were no licenses required; plant materials were gathered under the supervision and consent of the Faculty of Science at Alexandria University, as well as national guidelines, and all authors followed all local and national guidelines. All tests were carried out in accordance with the University of Alexandria lab guidelines. Ethics permission and agreement to participate. We have included in the techniques part the data on the identification of the plant materials.

## Consent for publication

Not applicable.

## CRediT authorship contribution statement

**Roufaida M. Elghobashy:** Writing – review & editing, Writing – original draft, Software, Resources, Project administration, Methodology, Investigation, Formal analysis, Data curation. **Shimaa A. Rashed:** Resources, Methodology, Formal analysis, Data curation. **Amal M. Fakhry:** Writing – original draft, Supervision, Resources, Project administration, Formal analysis, Data curation, Conceptualization. **Reham M. Mostafa:** Software, Funding acquisition, Formal analysis, Data curation. **Heba S. Essawy:** Visualization, Resources, Methodology, Funding acquisition. **Salama M. El-Darier:** Writing – original draft, Supervision, Resources, Investigation, Data curation, Conceptualization.

## Declaration of competing interest

The authors declare that they have no known competing financial interests or personal relationships that could have appeared to influence the work reported in this paper.
